# Comparative study of life satisfaction among patients with psoriasis versus healthy comparison group: the explanatory role of body image and resource profiles

**DOI:** 10.1007/s11136-020-02621-3

**Published:** 2020-09-04

**Authors:** Marcin Rzeszutek, Katarzyna Podkowa, Małgorzata Pięta, Daniel Pankowski, Sylwia Cyran-Stemplewska

**Affiliations:** 1 Faculty of Psychology, University of Warsaw, Stawki 5/7, 00-183 Warsaw, Poland; 2 Faculty of Psychology, University of Economics and Human Sciences in Warsaw, Okopowa 59, 01-043 Warsaw, Poland; 3 The Jan Kochanowski University, Żeromskiego 5, 25-369 Kielce, Poland

**Keywords:** Psoriasis, Life satisfaction, Body image, Resources, Latent profile analysis

## Abstract

**Purpose:**

The aim of the study was first to examine the heterogeneity of body image and resources, as described by the conservation of resources theory (COR), in a sample of psoriatic patients and explore whether heterogeneity within these variables explains the possible differences in levels of life satisfaction among the participants. Second, we aimed to investigate if life satisfaction level among the observed profiles of psoriatic patients, extracted on the basis of their body image and resources, differed from that of the healthy comparison group.

**Methods:**

The sample consisted of 735 participants, including 355 adults with a medical diagnosis of psoriasis and 380 healthy adults recruited from a non-clinical general population. Participants filled the Satisfaction with Life Scale, the Multidimensional Body-Self Relations Questionnaire and the COR evaluation questionnaire.

**Results:**

Latent profile analysis revealed four classes of psoriatic patients with different levels of resources and body image. The group with the highest level of resources and the most positive body image did not differ from the healthy comparison group regarding satisfaction with life. The group with the lowest level of resources and the most negative body image was characterized by the lowest satisfaction with life.

**Conclusions:**

The results of our study may change the simplifying trend that highlights the traditionally very poor well-being of psoriatic patients. Moreover, the discovery of specific profiles of these patients, which differ with regard to psychological variables, can lead to rethinking contemporary forms of psychological counselling in psoriatic patients.

Psoriasis is a chronic, inflammatory and incurable dermatological disease associated with visible and painful skin lesions and disfigurements, which may involve all parts of the body and significantly decrease patients’ well-being [2, 34]. Psoriasis affects men and women regardless of age or ethnicity and its worldwide prevalence varies between 0.91% in the United States to 8.5% in Norway [14]. Individuals suffering from psoriasis are at great risk of developing other chronic medical conditions such as cardiovascular diseases, hypertension, stroke or Crohn’s disease [1]. Moreover, this disease poses a major psychological burden related with experiences of constant embarrassment, stigma, social isolation and very poor self-esteem (see review, e.g. [24]). In particular, psoriatic patients display profound distortions in their body perception and negative body image impacts not only their psychological well-being, but also treatment adherence and treatment outcomes [15, 30, 36, 49]. The aforementioned factors can be related with mental health problems. For example, compared to the general population psoriatic patients are 40–90% more in danger of depression [25, 50] and anxiety [10, 12]. Thus, it is not a surprise that their subjective well-being is one of the worst compared to that of patients with other chronic or even life-threatening illnesses [34, 38]. Some authors found that the major issue associated with well-being among this patient group is the subjective perception of the severity of their illness, which is greatly shaped by psychosocial resources [44]. Furthermore, an increasing number of authors have underlined that psychological factors outweigh the role of medical variables illustrating the objective severity of the psoriasis as predictors of well-being in this patient group [26, 36]. Thus, in our study we focused on the role of subjectively assessed resources from the conservation of resources theory (COR; [17]), and their link to body image and life satisfaction in psoriatic patients. The first novelty of our study relates to the application of COR theory to illustrate the process of coping with psoriasis, as until now only the transactional model of stress and coping [27] has been used in this regard (e.g. [11, 12, 44]).

COR theory describes the sociocultural aspects of stress and coping and focuses on resources defined as things that the individual currently possesses and values (e.g. objects, states or conditions) or aims to achieve, maintain and protect in the future [17]. More specifically, this theory emphasizes the objective nature of stress simultaneously positing that although most resources are universally valued, their relative worth is likely to vary between people. Thus, the COR model proposes an important role for appraisal, but states that resources have both objective and subjective components. Until now, the majority of studies on Hobfoll’s theory have been carried out in non-clinical settings (e.g. [19, 20, 22, 48]) and much less is known about the application of this theory to the clinical environment, especially in patients struggling with chronic illness [8, 43]. For example, Dirik and Karanci [8] showed that loss of resources as described by COR can be an important predictor of illness-related distress (see depression and anxiety) among rheumatoid arthritis patients. In addition, Rzeszutek et al. [43] observed the role of family resources from the COR theory for better psychological functioning among females suffering from rheumatoid arthritis and females with breast cancer.

As far as psoriatic patients are concerned there are two particular reasons why COR theory is useful for illustrating how well or poorly they are coping with this disease. First, medical variables are very weakly related with the subjective well-being of psoriatic patients, i.e. the way these patients perceive their skin is much more important for their well-being than the objective severity of the psoriasis as assessed by clinical biomarkers [35, 39, 47]. In the constructive perception of skin symptoms, family resources are crucial [21]. Second, psoriasis has substantial repercussions on multiple aspects of the functioning of these patients ranging from family or intimate relationships to patients’ body image [16, 31, 33, 41]. Especially this latter variable, which is a multidimensional construct describing individual’s thoughts, beliefs, emotions and behaviours associated with one’s physical appearance, is shaped greatly by social and cultural factors [4]. COR theory illustrates the sociocultural aspects of stress and coping and resources as described by COR confer a symbolic value as they are essential to one’s self-image and identity [19]. However, till now the relationship between COR resources and body image in the clinical settings was noticed only among rheumatoid arthritis patients [37], so examining this relationship in case of psoriasis is an interesting research gap to fill.

There is an ongoing dispute on the process of the adaptation of subjective well-being (SWB) as consequence of experiencing stressful life events [7, 28], including also the case of coping with chronic illness [3, 42, 45]. A majority of authors showed the large empirical evidence for the “hedonic treadmill” model [29], i.e. “stability despite loss”, of well-being in reaction to life stress and adversity [9]. This latter pattern refers especially to the *tripartite model of subjective well-being* by Diener et al. [6, 7], and its cognitive component, i.e. life satisfaction, which was found to be a relatively stable, global evaluation of a person’s life [7]. The “stability despite loss” of life satisfaction was found also in aforementioned research on chronically ill patients [3, 45]. In our study, we conducted a comparative analysis of the life satisfaction level between the sample of psoriatic patients and healthy comparison group to indirectly check whether the aforementioned pattern may be present also in this particular, clinical sample. Comparative studies on this topic are very scarce and have provided mixed results, especially with regard to the variable of life satisfaction. While Solovan et al. [46] observed intuitively obvious, poorer life satisfaction among psoriatic patients, Reimus et al. [40] found no differences in life satisfaction level between these patients and healthy comparison group. The latter authors concluded that when individuals suffering from psoriasis are able to continue their usual daily activities and maintain an average day schedule thanks to family or partner support their painful skin symptoms do not diminish their life satisfaction.

Finally, to the best of our knowledge, contemporary studies on the psychological aspects of struggling with psoriasis have been based only on a *variable-centred approach*, which disregards the problem of the *heterogeneity* of participants within the studied variables (see reviews, e.g. [2, 24, 34]). In other words, those studies focused on identifying single sociomedical and/or psychological variables that were independently related to various aspects of well-being, coping or general functioning among these patients. Therefore, the application of a *person-centred perspective*, exploring the unique profiles of participants within the study variables, is the third novelty of our research.

## Current study

Taking the above-mentioned research gaps into consideration, the main aim of our study was two-fold. First, we wanted to examine the heterogeneity of body image and resources in the sample of psoriatic patients and explore whether heterogeneity within these variables could explain the possible differences in levels of life satisfaction among participants, while controlling for their sociomedical data. Second, we aimed to investigate if the observed profiles of psoriatic patients, extracted on the basis of their body image and resources, differed from the healthy comparison group with regard to the level of life satisfaction. To the best of our knowledge, there are no studies conducted among psoriatic patients that may be useful for us as a direct source of research hypotheses in the case of this special study design. Thus, we mainly employed an exploratory approach in our study. However, based on some existing studies within a different methodological framework (see above, e.g. [2, 10, 24, 34]), we expected that our clinical sample would be heterogeneous in terms of body image and resources and that the observed profiles of these psychological variables would be differently related to levels of life satisfaction within the group of psoriatic patients, while controlling for sociomedical covariates. Moreover, we assumed that psoriatic patients would declare, on average, lower levels of life satisfaction and a more negative body image and would assess their psychosocial resources as worse compared to the healthy comparison group. We also hypothesized that participants from various profiles, extracted on the basis of their body image and resources, would declare different levels of life satisfaction compared with the healthy comparison group.

## Method

### Participants and procedure

The sample was comprised of 355 adults with a medical diagnosis of psoriasis and 380 healthy adults (without any chronic illnesses) recruited from a non-clinical population. The psoriatic patients were recruited both from among patients hospitalized in two dermatology clinics in Poland, where they filled out traditional, paper-and-pencil questionnaires, and from members of the Union of Associations of Patients with Psoriasis in Poland, who filled out an online inventory distributed via social media. Specifically, the online part of the study was conducted because of the possibility of reaching patients all over Poland; in our study we did not want to limit ourselves only to clinics in large cities. Working online allowed us to recruit people from small towns, as well as individuals who do not have an active disease at the moment and do not require hospitalization. Importantly, in order to be a member of the Union of Associations of Patients with Psoriasis in Poland the patient should submit a medically confirmed diagnosis of psoriasis.

As far as the first mode of recruitment is concerned, 105 of the participants with psoriasis were patients of the Dermatology Clinic at the Military Institute of Medicine in Warsaw or the Dermatology Clinic of the Provincial Integrated Hospital in Kielce. Specifically, out of 180 patients eligible for the study in these two clinics, 105 were recruited and agreed to filled out questionnaires (58%), 36 declined (20%), and 39 (22%) completed the inventories with a very high level of missing data, which precluded including them in the statistical analysis. In the online mode, 250 psoriatic patients agreed to fill out the study questionnaires. In both modes of the participants’ recruitment the eligibility criteria included being 18 years of age or older and having a medical diagnosis of psoriasis for at least 1 year. The exclusion criteria included a recent outbreak of psoriasis, i.e. having had psoriasis for <1 year. In both modes of participant recruitment the study subjects were given informed consent and participated in the study voluntarily, as there was no remuneration in exchange for participation.

The healthy comparison group was recruited among students from various Warsaw universities and this group filled out the study questionnaires online via social media. The both inclusion and exclusion criteria encompassed being 18 years of age or older, no history or presence of chronic medial illnesses and willingness to take part of this study. In particular, this comparison sample was recruited after psoriatic patient cohort was collected, because we tried to maintain purposeful sampling in order to obtain comparison group with demographic characteristics similar to those of our clinical sample. The study was approved by the local ethics commission.

Table 1 summarizes the sociomedical variables in both groups of participants with values of statistical test. Categorical variables were tested with the use of Pearson’s chi-squared test of independence. Participants’ age was tested with the use of Student’s *t* test for independent samples.

**Table 1:** Sociomedical variables in the studied sample of psoriatic patients (*N* = 355) and healthy comparison group (*N* = 380)

Variable	*N* (%) Psoriasis	*N* (%) Healthy comparison group	Significance test
Gender			
Male	83 (23.4%)	58 (15.3%)	*χ*^2^(1) = 7.80, *p* < *.*001
Female	272 (76.6%)	322 (84.7%)	
Age in years (M ± SD)	23.42 ± 12.42	29.52 ± 7.07	*t*(553.12) = − 16.79, *p* < *.*001
Marital status			
Married	260 (73.2%)	204 (53.7%)	*χ*^2^(1) = 30.15, *p* < *.*001
Single	95 (26.8%)	176 (46.3%)	
Education			
Elementary	8 (2.3%)	6 (1.6%)	*χ*^2^(3) = 89.99, *p* < *.*001
Vocational	22 (6.2%)	3 (.8%)	
Secondary	141 (39.7%)	279 (73.4%)	
Higher education	184 (51.8%)	92 (24.2%)	
Employment			
Full employment	237 (66.8%)	171 (45.0%)	*χ*^2^(3) = 77.77, *p* < *.*001
Unemployed	86 (24.2%)	199 (52.4%)	
Illness allowance	9 (2.5%)	10 (2.6%)	
Retired	23 (6.5%)	0 (0%)	
Village, small town up to 20 thousand residents	103 (29.0%)	62 (16.3%)	*χ*^2^(4) = 88.31, *p* < *.*001
City 21 to 100 thousand residents	69 (19.4%)	45 (11.8%)	
City 101 to 500 thousand residents	71 (20.0%)	25 (6.6%)	
City over 500 thousand residents	109 (30.7%)	244 (64.2%)	
Lack of permanent residence	3 (0.8%)	4 (1.1%)	
Type of psoriasis			
Ordinary	256 (72.1%)	–	
Pustular	38 (10.7%)	–	
General	3 (0.8%)	–	
Psoriatic arthritis	36 (10.1%)	–	
Other	17 (4.8%)	–	
Missing data	5 (1.4%)	–	
Years of diagnosis (M ± SD)	16.66 ± 12.27	–	

*M* mean, *SD* standard deviation, *χ*^*2*^ Pearson’s Chi-squared test of independence, *t* Student’s *t* test for independent samples

*p*, statistical significance

The percentages of males, married participants, participants with higher education, and participants with full employment were higher in the clinical sample. The percentage of participants living in cities with over 500 thousand residents was higher in the healthy control group. Sociomedical variables that differed between the two groups and were significantly related to the explained variable were controlled for in the subsequent analysis.

### Measures

Life satisfaction was assessed with the Satisfaction with Life Scale (SWLS; [6]) in a Polish adaptation. SWLS consists of five items, respondents assess each item on a seven-point scale ranging from one (*strongly disagree*) to seven (*strongly agree*). For example: *I am satisfied with my life*; *So far I have gotten the important things I want in life*. Thus, a higher total score on this scale indicates a higher level of life satisfaction. McDonald’s omega [32] coefficient for the SWLS in the sample of psoriatic patients was .87 and in the healthy comparison group was .90.

Participants’ body image was evaluated with the Multidimensional Body-Self Relations Questionnaire (MBSRQ) created by Cash [4] and adapted to Polish. The MBSRQ consists of ten scales describing several aspects of body image: the appearance evaluation scale, the appearance orientation scale, the fitness evaluation scale, the fitness orientation scale, the health evaluation scale, the health orientation scale, the illness orientation scale, the body-areas satisfaction scale, the overweight preoccupation scale and the self-classified weight scale. The particular items of this inventory are as following: *I always pay attention to what I look like before leaving the house*, *I am careful to buy clothes that will allow me to look my best*. The higher the results in each subscale, the more positive the particular body image aspect. In the clinical sample, subjects were instructed to focus on possible changes in their body image as a results of their disease experiences. McDonald’s omega coefficients for the MBSRQ subscales varied between .67 to .76 in the clinical sample and .76 to .88 in the healthy comparison group.

To assess the level of COR resources, we used the COR evaluation questionnaire (COR-E; [18]) in a Polish adaptation. The COR-E rated the extent to which the patients possessed several resources, such as hedonistic and vital resources, spiritual resources, family resources, economic and political resources, and power and prestige resources. Particular items of this inventory relate to e.g. *Having material security in old age*; *Employment assurance; Family support*. In the clinical sample, subjects were instructed to focus on their level of resources in the context of their disease experiences. McDonald’s omega coefficients for the COR subscales varied between .84 to .94 in the clinical sample and .78 to .93 in the healthy comparison group.

Finally, only among the psoriatic patients the disease-specific quality of life was assessed using the *Physical Symptoms Scale* included in Skindex-29 [5] in the Polish adaptation. This scale assesses seven dermatological symptoms on a 5-point rating scale ranging from *never* to *all the time;* higher scores mean more severe symptoms. McDonald’s omega coefficient for Skindex-29 in the sample of psoriatic patients was .90.

## Data analysis

Table 2 presents descriptive statistics for the analysed variables, i.e. the mean values, standard deviations, skewness and kurtosis measures and McDonald’s omega coefficients of reliability. None of the measures of skewness or kurtosis exceeded the value of 1 or − 1; therefore normal distribution of the analysed variables was assumed, which is the necessary condition for the analysis of variance.

**Table 2:** Descriptive statistics for analysed variables (*N* = 735)

Variables	*M*	SD	*S*_p_	*K*_p_	*S*_c_	*K*_c_	*ω*
1. Hedonistic and vital resources	34.82	10.83	− .35	− .09	− .49	− .26	.91
2. Spiritual resources	23.09	5.83	− .07	− .43	− .35	− .03	.83
3. Family resources	27.56	10.24	.34	− .62	− .54	− .51	.95
4. Economic and political resources	21.56	7.68	.09	− .59	− .28	− .18	.87
5. Power and prestige	11.73	5.50	.19	− .54	− .07	− .39	.82
6. Appearance evaluation	3.19	.87	− .33	− .83	− .25	.41	.88
7. Appearance orientation	3.20	.55	− .18	− .18	.22	.63	.77
8. Fitness evaluation	3.25	.93	− .28	− .70	− .14	− .24	.66
9. Fitness orientation	3.12	.74	− .04	− .71	.38	.96	.86
10. Health evaluation	3.23	.73	− .31	− .55	− .12	.42	.77
11. Health orientation	3.04	.67	− .07	.13	− .01	− .19	.78
12. Illness orientation	3.24	.75	.01	− .40	− .05	− .32	.71
13. Overweight preoccupation	2.63	.90	.30	− .68	.09	.04	.69
14. Body-Areas Satisfaction	3.12	.71	− .27	− .33	.05	.12	.82
15. Self-classified Weight Scale	3.22	.84	.45	.79	− .25	− .10	68
16. Satisfaction with Life	18.06	6.26	− .09	− .59	.24	− .01	.89
17. Skindex Physical Symptoms Scale	21.30	5.13	–	–	− .12	.23	.90

*M* mean value, *SD* standard deviation, *S*_*p*_ Skewness in the clinical group, *K*_*p*_ Kurtosis in the clinical group, *S*_*c*_ Skewness in the comparison group, *K*_*c*_ Kurtosis in the comparison group, *ω* McDonald’s *ω* reliability coefficient

The data analysis consisted of three consecutive steps. First, we examined the associations between the sociomedical data and the dependent variable, i.e. satisfaction with life. Second, we extracted four classes of respondents from the clinical group that differed regarding their conjoint profile of resources and body image. Finally, we examined the differences between the extracted classes and the comparison group in terms of satisfaction with life. Power analysis proved that with four classes extracted the sample size of 355 patients with psoriasis let us detect statistically significant differences regarding satisfaction with life of effect size equal to .18 in terms of Cohen’s *f* effect size measure. We assumed statistical power of .80 and conventional .05 point of statistical significance.

Associations between the sociomedical data and satisfaction with life were examined with the use of independent samples, *t* test and Pearson’s correlation coefficient. There was no statistical difference between men and women, *t*(733) = .07, *p* > .05. Satisfaction with life did not correlate with participants’ age, *r*(733) = − .01, *p* > .05. However, satisfaction with life was significantly higher in the group of married participants compared to singles, *t*(733) = 3.29, *p* < .01. The mean value of satisfaction with life was equal to 18.63 (SD = 6.13) in the group of married participants and to 17.07 (SD = 6.37) in the group of single participants. The mean value of satisfaction with life was equal to 18.79 (SD = 6.22) in the group of participants with higher education and was higher than mean and equal to 17.62 (SD = 6.25) in the group of participants without higher education *t*(733) = − 2.46, *p* < .05. Participants with full employment did not differ from participants unemployed or retired, *t*(733) = − 1.44, *p* > .05. The mean value of satisfaction with life was equal to 18.68 (SD = 6.46) in the group of participants living in cities with over 500,000 residents and was higher than mean and equal to 17.48 (SD = 6.01) in the group of participants living in minor localities, *t*(733) = − 2.61, *p* < .01. Satisfaction with life did not correlate with years of diagnosis, *r*(353) = .014, *p* > .05; however it correlated negatively with the Skindex score, *r*(355) = − .170, *p* < .01.

In the next step, latent profile analysis was executed in order to estimate distinct profiles and extract different subgroups of respondents differing in regard to resources and body image. We analysed five types of resources and ten indicators of body image. According to the values of Aikake information criterion (AIC) and Bayesian information criterion (BIC), the model with the best fit was the model with equal variances and covariances fixed to zero and with four extracted classes with four distinctive profiles. The values of fit statistics were equal to AIC = 13,933.15 and BIC = 14,235.18 with entropy value equal to .82 where values > .07 mean acceptable classification accuracy [23]. Values of fit indices for all models tested are provided in Table 3.

**Table 3:** Fit indices and entropy values for models tested in latent profile analysis

Classes	AIC	BIC	Entropy
1	15,156.67	15,272.84	1
2	14,346.98	14,525.1	.87
3	14,058.64	14,298.71	.85
4	13,933.15	14,235.18	.82
5	13,933.74	14,297.72	.83
6	13,978.41	14,304.34	.84

**Fig. 1:**
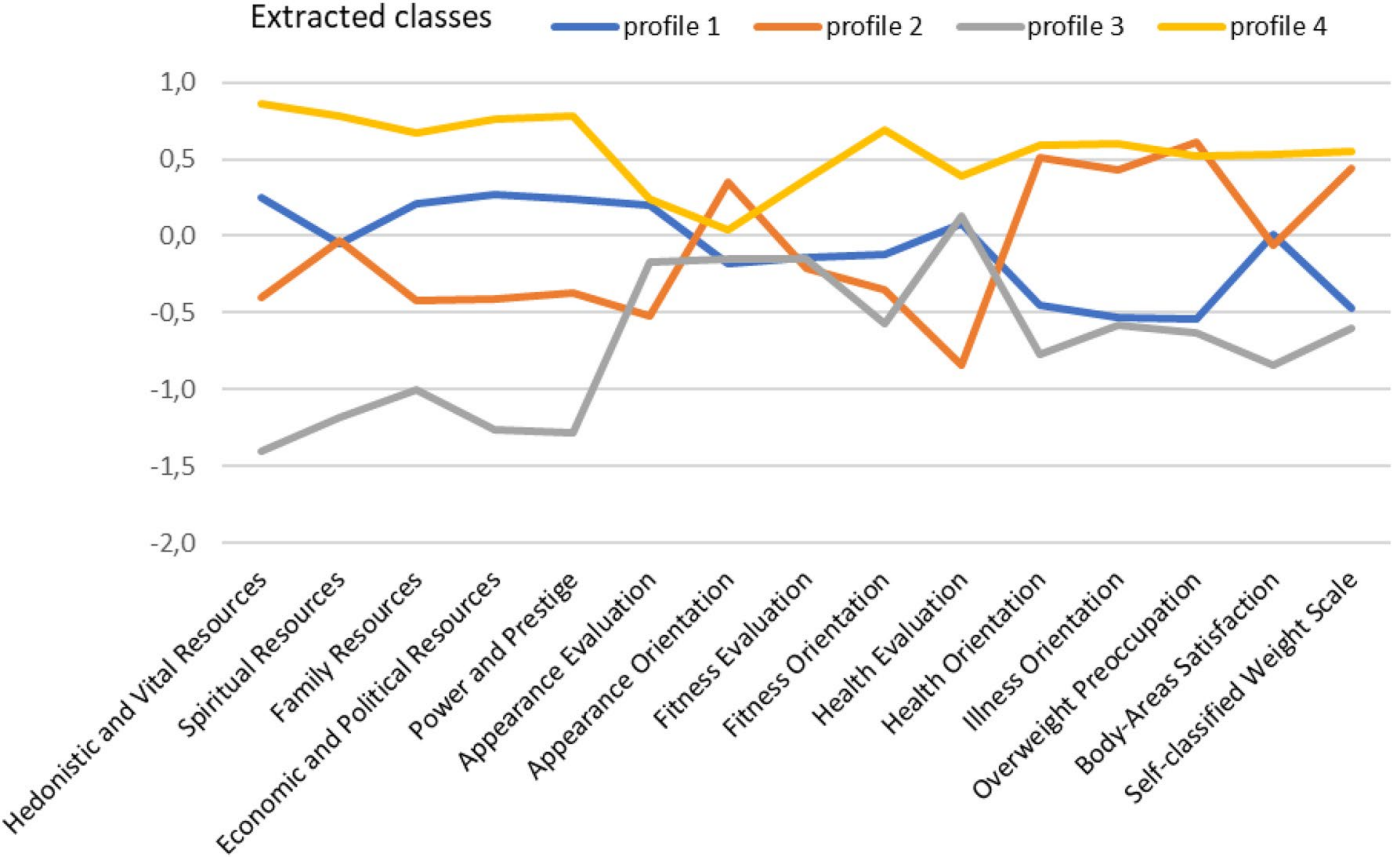
Profiles of resources and body image acquired in the group of psoriatic patients

Figure 1 presents the mean values of the standardized variables in the acquired classes.

According to results of MANOVA the four classes differed in terms of resources and body image, *F*(45,1017) = 21.84, *p* < .001, *η*^2^ = .49. This applies to Hedonistic and Vital Resources, *F*(3,351) = 213.40, *p* < .001, *η*^2^ = .65, Spiritual Resources, *F*(3,351) = 94.43, *p* < .001, *η*^2^ = .45, Family Resources, *F*(3,351) = 70.86, *p* < .001, *η*^2^ = .38, Economic and Political Resources, *F*(3,351) = 131.59, *p* < .001, *η*^2^ = .53, Power and Prestige, *F*(3,351) = 138.23, *p* < .001, *η*^2^ = .54, Appearance Evaluation, *F*(3,351) = 11.66, *p* < .001, *η*^2^ = .09, Appearance Orientation, *F*(3,351) = 4.77, *p* < .01, *η*^2^ = .04, Fitness Evaluation, *F*(3,351) = 7.51, *p* < .001, *η*^2^ = .06, Fitness Orientation, *F*(3,351) = 57.87, *p* < .001, *η*^2^ = .33, Health Evaluation, *F*(3,351) = 28.15, *p* < .001, *η*^2^ = .19, Health Orientation, *F*(3,351) = 57.87, *p* < .001, *η*^2^ = .33, Illness Orientation, *F*(3,351) = 49.26, *p* < .001, *η*^2^ = .30, Overweight Preoccupation, *F*(3,351) = 56.13, *p* < .001, *η*^2^ = .32, Body-Areas Satisfaction, *F*(3,351) = 32.70, *p* < .001, *η*^2^ = .22, and Self-classified Weight Scale, *F*(3,351) = 42.76, *p* < .001, *η*^2^ = .27. According to Gabriel post-hoc test all classes differed in terms of Hedonistic and Vital Resources, *p* < .001, Family Resources, *p* < .001, Economic and Political Resources, *p* < .001, and also in terms of Power and Prestige, *p* < .001. All classes with the exception of the difference between profile 1 and profile 2 differed in terms of Spiritual Resources, *p* < .001, and Body-Areas Satisfaction, *p* < .001. All classes with the exception of the difference between profile 1 and profile 3 and the difference between profile 2 and profile 4 differed in terms of Health Orientation, *p* < .001, Illness Orientation, *p* < .001, Overweight Preoccupation, *p* < .001, and Self-classified Weight Scale, *p* < .001. There were statistically significant differences in terms of Appearance Evaluation between profile 2 and profiles 1 and 4, *p* < .001, and also between profiles 3 and 4, *p* < .05. There were statistically significant differences in terms of Appearance Orientation between profile 2 and profiles 1, *p* < .01 and 3, *p* < .05. Profile 4 differed in terms of Fitness Evaluation from profile 1, profile 2, and profile 3, *p* < .01. Profile 4 also differed in terms of Fitness Orientation from profile 1, profile 2, and profile 3, *p* < .01, but there was also statistically significant difference in terms of Fitness Orientation between profiles 1 and 3, *p* < .01. Profile 2 differed in terms of Fitness Evaluation from profile 1, profile 3, and profile 4, *p* < .001.

In the first class the acquired profile was characterized by an average level of resources and an average body image assessment (*profile 1*). In the second class the acquired profile was characterized by an average level of resources and a higher-than-average evaluation of specific body image subscales (orientation on appearance, health, disease and absorption of weight; *profile* 2). In the second class the acquired profile was characterized by a low level of resources and poor body image assessment (*profile* 3); finally, the fourth class revealed a high resources evaluation and the most favourable outlook on body image (*profile 4*).

**Fig. 2:**
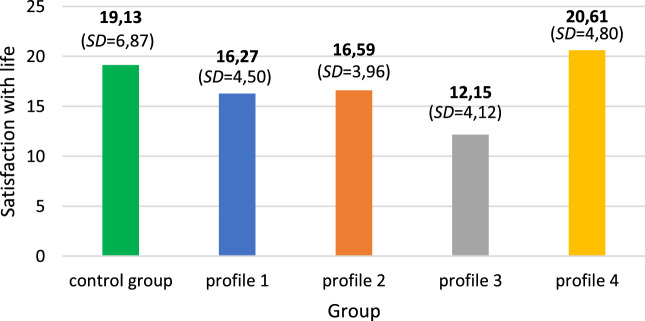
Mean values of satisfaction with life in the extracted classess of psoriatic patients compared to the healthy comparison group

The extracted classes of psoriatic patients were compared to each other and to the comparison group. Taking into account the sociomedical data correlating with satisfaction of life, analysis of covariance was performed. Marital status, education, place of residence and Skindex score were included in the statistical model as covariates. Differences between the groups were statistically significant: *F*(4,726) = 18.65, *p* < .001, *η*^2^ = .09. According to the value of contrast test, the clinical sample significantly differed from the comparison group: *t*(636.96) = − 6.45, *p* < .001. The mean value of satisfaction with life was lower in the group of psoriatic patients (*M* = 16.90; SD = 5.29) than in the comparison group (*M* = 19.13; SD = 6.26). However, a post-hoc comparison based on the Games-Howell test revealed this difference to be spurious. Figure 2 presents the mean values of satisfaction with life in the extracted classes and in the comparison group.

According to the post-hoc test, satisfaction with life in the comparison group differed from the classes with profile 1, *p* < .001; profile 2, *p* < .01; and profile 3, *p* < .001. However it did not differ from profile 4, *p* > .05. Satisfaction with life in the comparison group and in the class with the high level of resources and positive body image was similar. Satisfaction of life in the class with profile 1 did not differ from the class with profile 2, *p* > .05, even though body image in the class with profile 2 was slightly better. The class with profile 3 significantly differed from all of the other classes and from the comparison group (all *p* < .001). The most negative body image and the lowest level of resources was associated with the least satisfaction with life.

## Discussion

The results of our study were consistent with the first research hypothesis, as our clinical sample of psoriatic patients were heterogenous with regard to their body image assessments and resources evaluation, which were differently related to their levels of life satisfaction. More specifically, we observed four profiles of participants with an average level of resources and an average body image assessment (*profile 1*), an average level of resources and a higher-than-average evaluation of specific body image subscales (orientation on appearance, health, disease and absorption of weight; *profile 2*), a low level of resources and poor body image assessment (*profile 3*), and finally, a high level of resources and the most favourable outlook on body image (*profile 4*). When comparing these four profiles with each other we found the biggest discrepancy in life satisfaction between two extreme profiles with respect to resources and body image assessment. Specifically, the highest level of satisfaction with life was observed in the fourth profile, while the lowest level of life satisfaction was seen in the third profile. Significantly, medical variables such as the type of psoriasis, the duration of struggle with the disease and the intensity of physical symptoms were unrelated to life satisfaction among our participants. This result can be interpreted in light of the above-mentioned studies pointing to the fact that psychosocial variables, including especially positive self-and body image, outweighed the role of clinical biomarkers illustrating the objective severity of psoriasis as predictors of well-being in this patient group [15, 26, 36]. For example, the most popular and widely used tool to assess the severity of psoriasis, the Psoriasis Area and Severity Index (PASI), has been found to be very weakly associated with the well-being of these patients in many studies (see review [2, 24]).

Moreover, we observed one more interesting issue. Namely, we noticed that life satisfaction level in profiles 1 and 2, which consisted of participants with various attitudes to their body image (see focus on appearance, health, illness and preoccupation with weight) and a similar, average level of resources, was the same. Several studies showed the vicious circle mechanism of negative body image on psychological functioning among psoriatic patients, i.e. negative body perception fosters negative mood and further distorts illness severity perception, which subsequently makes the body image even worse etc. [16, 33, 41]. Conversely, many authors found that positive body image may facilitate more effective coping with physical symptoms of psoriasis and enhance well-being in these patients [15, 36]. However, our above-mentioned finding may suggest that a positive outlook on body image alone is not enough to sustain a high level of life satisfaction when a patient does not perceive an availability of resources in his or her close environment. Although until now no research on COR theory has been conducted among psoriatic patients, our results may be in line with other studies highlighting the need to assess the multidimensional influence of psoriasis on different aspects of the functioning of these patients [12, 30, 31].

Our second, most intuitive hypothesis was also confirmed, as we observed that psoriatic patients declared, on average, lower levels of life satisfaction, more negative body image and lower levels of psychosocial resources compared to the healthy comparison group. Dozens of studies have shown that psoriasis significantly diminishes patients’ well-being and is related to mental health problems not only to the general population, but also to other chronic diseases (see e.g. reviews [10, 25]). However, these studies focused only on a *variable-centred approach*, i.e. the identification of single sociomedical and/or psychological variables that were independently related to various aspects of well-being in these patients. Perhaps this unitary methodological approach was responsible for the traditional, gloomy and probably simplifying trend pointing to the almost always worse well-being of psoriatic patients compared to the general population [34]. In our study we believed that the application of a *person-centred perspective* would provide a more accurate picture of life satisfaction and its determinants in these patients.

In accordance with these expectations we observed that while comparing four profiles of psoriatic patients to the comparison group, individuals from profiles 1, 2 and 3 declared lower life satisfaction than subjects from the comparison group. However, patients from the fourth profile did not differ from the comparison group in the level of life satisfaction and had even higher levels of resources and a better body image assessment than the healthy comparison group. Our findings may shed new light on the determinants of well-being [2, 49] and life satisfaction [40, 46] among psoriatic patients. Particularly, despite the great psychological and physical burden of this disease, patients with psoriasis may sustain the same life satisfaction as people from the general population, but this requires a higher perceived availability of psychosocial resources and better outlook on body image than that of the healthy comparison group. This finding will obviously require replication in a larger sample of psoriatic patients, but may indicate on two important issues. First, the traditional methodological approach (see *variable-centred*) to assessing various aspects of the psychological well-being of psoriatic patients precludes obtaining a true and comprehensive picture of this phenomena in this disease [34]. Second, psychological interventions for psoriatic patients should concentrate less on patient education and self-management in coping with psoriatic symptoms only (see [51]), and could be more grounded in counselling focusing on building psychosocial resources in the patient’s closest community and working on a more positive body image in particular. This is in line with the most recent interventions for chronically ill patients proposed by authors represented the positive psychology, highlighting the need for building patients’ positive resources, especially related to their positive self-image [13].

## Strengths and limitations

This study has several strengths, including the large sample of psoriatic patients and the comparative analysis with a healthy comparison group using an innovative methodological design. Nevertheless, it is vital to point out some limitations of our research. First of all, the cross-sectional framework of this study does not allow for causal interpretations of the obtained findings. Second, the clinical group was heterogonous with regard to the diagnosis of psoriasis, as well as its duration and the severity of its symptoms. Future studies should focus on a more homogenic sample of psoriatic patients with regard to the medical variables. In addition, although we tried to recruit the comparison group with high similarity in socio-demographic characteristics to the clinical sample, we did not entirely succeed in this task. Finally, it should be underscored that in this study we focused only the one side of resources, i.e. their subjective appraisal, and we did not attempt to parallelly assess also the objective level of the participants’ resources.

## Conclusions

Despite its limitations, our study addressed significant research gaps in the literature on psychological well-being among psoriatic patients. Specifically, from the theoretical point of view, the *person-centred* framework may provide insight much above that which can be gained using *variable-centred* methods and thus change the simplifying trend highlighting the traditionally very poor well-being of this patient group. In addition, from practical perspective, approaching psychological counselling based on the discovery of specific profiles of psoriatic patients, which differ with regard to sociomedical and psychological variables, may address the unique needs of these patients. In other words, one should remember that beyond the *clinical label*, known as psoriasis, there is always an individual human who should not be treated as a homogenic representative of the disease.
